# Decreased Amount of Supporting Alveolar Bone at Single-Rooted Premolars Is Under Estimated by 2D Examinations

**DOI:** 10.1038/srep45774

**Published:** 2017-04-03

**Authors:** Hsiang-Hsi Hong, Chung-Chieh Chang, Adrienne Hong, Heng-Liang Liu, Yen-Li Wang, Shih-Hao Chang, Tzung-Hai Yen

**Affiliations:** 1 Department of Periodontics, Chang Gung Memorial Hospital and Chang Gung University, Linkou; School of Dental Technology, College of Oral Medicine, Taipei Medical University, Taipei, Taiwan; 2 Department of Periodontics, Chang Gung Memorial Hospital, Linkou, Taiwan; 3 California Northstate University, College of Medicine, Elk Grove, CA, United States; 4 Instrument Department, Chang Gung Memorial Hospital, Linkou, Taiwan; 5 Department of Periodontics, Chang Gung Memorial Hospital, Taoyuan, Taiwan; 6 Department of Periodontics, Chang Gung Memorial Hospital and Chang Gung University, Linkou, Taiwan; 7 Department of Nephrology, Chang Gung Memorial Hospital and Chang Gung University, Linkou, Taiwan

## Abstract

The purpose of this study was to relate the proportions of bone-supported root length of a 2D view into the amount of a 3D bone-attached root surface area (BA-RSA) by using a dental laser scanner examination. White-light 3D scanning technology was used to probe 36 maxillary and 35 mandibular single-rooted premolars. The bone-supported height (BSH) and BA-RSA at designated levels (95–25%) were compared using statistical *t* tests. The 100% BSH and BA-RSA of the maxillary/mandibular premolars were 12.6 ± 1.60 mm/13.45 ± 1.47 mm (*p* < 0.05) and 220.78 ± 35.31 mm^2^/199.51 ± 26.33 mm^2^ (*p* < 0.01), respectively. Approximately 79–80%, 59–60%, and 35–36% premolars 2D BSH remained in comparison to 75%, 50%, and 25% 3D BA-RSA preservation, respectively. However, corresponding to a 75%, 50%, and 25% 2D BSH reserve, premolars retained 67–68%, 39–41%, and 15–17% 3D BA-RSA, respectively. When taking 1.0 mm connective tissue attachment into account, 60% 3D BA-RSA and 50% 2D BSH loss were noted at the 5.1–5.4 mm clinical attachment level. Assigning a periodontal prognosis and determining the severity of periodontitis for premolars with alveolar bone loss based on 3D’s or 2D’s measurement is inconsistent.

Numerous factors contribute to the prognosis of periodontal treatment. Anatomic factors including short or tapered roots, cervical enamel projection, enamel pearls, bifurcation ridges, root concavities, developmental grooves, root proximity, furcation involvement, and tooth mobility may affect the treatment outcome for a specific tooth^1^. By contrast, the characteristics and quantity of residual periodontal attachment are crucial to determining the dental prognosis of individuals with periodontitis^2^. For example, when a tooth loses more than 50% of its periodontal attachment, it could be classified as having a questionable prognosis^1^,^3^,^4^. The severity and prognosis of teeth, such as pier abutment teeth, with periodontal destruction could be inconsistent when the amount of periodontal support is evaluated using either 3D or 2D approach.

To evaluate the prognosis of a specific type of tooth, such as a premolar, periodontists assess the crown–root ratio by using a 2D periapical X-ray film coupled with a clinical attachment level (CAL) by measuring the probing attachment level. The roots of human teeth present a tapered but also complex anatomic morphology (i.e., the radius at a cross-section varies when measured from the center of the root to the facial, lingual, mesial, or distal root surface at different corono-apical levels). Scholars have investigated the quantitative relationship between the amount of residual periodontal attachment and total root surface area of one tooth with regard to periodontium loss^5^,^6^,^7^,^8^. However, the differentiated information regarding the percentage of maximal bone support evaluated using a 2D view compared with that using a 3D image remains limited. The authors, therefore, hypothesized that comparing the bone-supported length (2D) and the bone-attached surface area (3D) at specific locations on the root from the apex to the cementoenamel junction (CEJ) required further examination.

Next-generation dental laser scanners (e.g., DentSCAN, Delcam, Birmingham, UK) use white-light 3D technology to capture data accurately up to 20 μm. Dental laboratories combine such scanners with precise calibration and articulation equipment to achieve high-precision scans designed for all types of dental restoration, ranging from simple copings to anatomical crowns and bridges for both in-house production and manufacturers at milling centers. However, dental laser scanning has not been able to clearly identify the association between periodontal attachment loss and root surface area (RSA) of the human tooth.

In the present study, the bone-supported height (BSH), accorded with periapical X-ray film findings, was characterized as a linear measurement of bone-supported root length from the apex to the CEJ. The linear–root ratio, presented in percentage, is the maximal bone-supported root length over the total root length in a 2D view at a specific level on the root from the apex to the CEJ. The bone-attached root surface area (BA-RSA) presented as an amount and as a percentage of bone-attached root surface area at an estimated level on the root from the apex to the CEJ over the total root surface area in a 3D view. Furthermore, the concept of biologic width, including 1.07 mm of connective tissue plus 0.97 mm of the epithelium, was essential in differentiating the inconsistency among CAL, BA-RSA, and BSH in this survey^9^.

The purpose of this study was to use a dental laser scanner to survey single-rooted premolars and to compare the BA-RSAs assessed by a 3D method with the BSHs evaluated by imitating a traditional 2D view. The difference in periodontal attachment loss between 2D and 3D views was explored, and the possible associations of present results with the periodontal prognosis defined by previous studies were also discussed.

## Results

**Figure 1:**
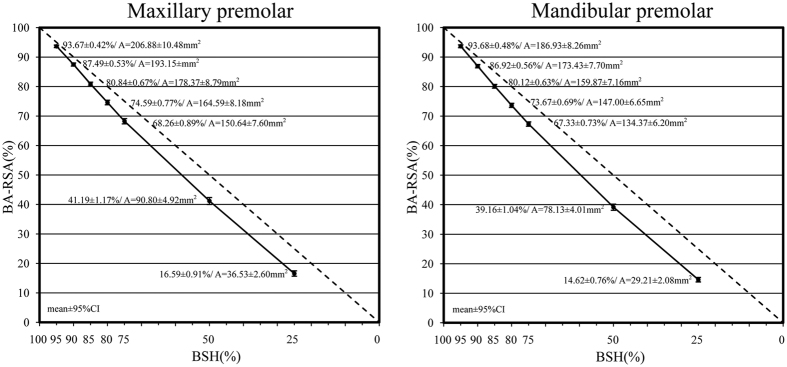
Amount of 3D BA-RSA at the evaluated 2D BSH levels. One-sample *t* test was used to compare the 3D BA-RSAs to the evaluated 2D BSHs: Mean values and 95% confidence intervals (95% CI): H0 (*p* ≥ 0.01) or H1 (*p* < 0.01).

**Figure 2:**
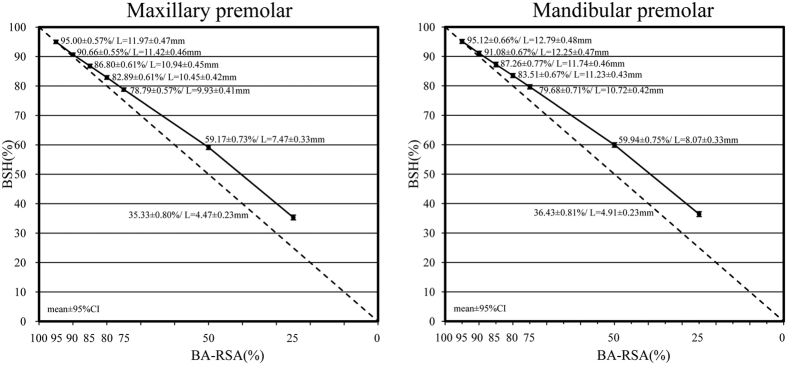
Corresponding 2D BSH levels to the assessed 3D BA-RSAs. One-sample *t* test was used to compare the 2D BSH levels to the appraised 3D BA-RSAs: Mean values and 95% confidence intervals (95% CI): H0 (*p* ≥ 0.01) or H1 (*p* < 0.01).

The collected premolars were surveyed. The resulting data are shown in Tables 1 and 2 and Figs 1 and 2.

### Comparison of BA-RSA and BSH at examine levels

The results revealed that the average BA-RSA of the maxillary and mandibular premolars were 220.78 ± 35.31 mm^2^ (range from 162 to 309 mm^2^) and 199.51 ± 26.33 mm^2^ (range from 151 to 279 mm^2^) respectively (*p* = 0.005). Comparisons showed that the BA-RSA proportions did not correspond to the BSH proportions at the levels of 90% to 25% for the maxillary and mandibular premolars (H1, *p* < 0.01, Fig. 1). The average root lengths of the maxillary and mandibular premolars were 12.60 ± 1.60mm (range from 8.9 to 16.1 mm) and 13.45 ± 1.47 mm (range from 10.6 to 17.0 mm) respectively (*p* < 0.05). The root lengths at the 80%, 75%, 50%, and 25% evaluated 2D BSH levels were inconsistent with the root lengths at the 80%, 75%, 50%, and 25% 3D BA-RSA assessed levels for the maxillary premolars. The root lengths at 85%, 80%, 75%, 50%, and 25% 2D BSH levels were inconsistent with the root lengths at the 85%, 80%, 75%, 50%, and 25% 3D BA-RSA levels for the mandibular premolars (Figs 1 and 2; H1, *p* < 0.05). Similar 3D BA-RSA and 2D BSH root lengths were only presented at the coronal 10% for mandibular premolars and 15% for maxillary premolars, respectively (H0, *p* ≥ 0.01, Fig. 2).

### Comparison of the BA-RSA significance at the evaluated BSH levels

In general, at the coronal 25% 2D BSH, both maxillary and mandibular premolars did not reveal significant differences in 3D BA-RSA percentages between each of the 5% segment. No significant difference was also noted between 100–87.5% and 87.5–75% 2D BSH levels for either premolar. Observing the 3D BA-RSA percentage changes for the four 25% 2D BSH levels (100–75%, 75–50%, 50–25%, and 25–0%), all the subsequent 25% BSH levels (e.g. 100–75% vs. 75–50%, 75–50% vs. 50–25%, and 50–25% vs. 25–0%) displayed significant BA-RSA percentage differences for both the maxillary and mandibular premolars (*p* < 0.01, Table 1).

### Comparison of the BSH significance at various BA-RSA levels

At the coronal 25% 3D BA-RSA, both the maxillary and mandibular premolars would reveal significant differences in 2D BSH percentages at each 5% levels (*p* < 0.01); however, the sequential 5% 3D comparison showed no significant difference. The 2D BSH percentages at the following analyzed levels were not distributed according to the corresponding maxillary premolars’ 3D BA-RSAs: 100–87.5% vs. 87.5–75%, 100–75% vs. 75–50%, 75–50% vs. 50–25%, or 50–25% vs. 25–0%. Neither were those of the mandibular premolars: 100–87.5% vs. 87.5–75%, 75–50% vs. 50–25%, or 50–25% vs. 25–0% (*p* < 0.01, Table 2).

## Discussion

In the current study, the maxillary and mandibular premolars differed significantly in root length and total RSA (*p* < 0.05). Mandibular premolars possess less varied anatomic morphologies and have a longer root length compared to maxillary premolars. A wider buccolingual dimension^10^ and a remarkable interproximal concave may explain a larger BA-RSA occurrence at the maxillary premolars.

The decreased amounts of 3D RSAs at evaluated 2D levels confirmed a taper pattern of human premolar roots, which narrowing did not follow a constant slope (Fig. 1). To support the indication of applying the proportion of 2D BSH levels to determine the amount of 3D BA-RSA, coronal 5% 3D BA-RSAs can only be comparably evaluated according to the coronal 5% 2D BSH levels for both maxillary and mandibular premolars (Fig. 1, H0). Generally, the amount of 3D BA-RSAs at two subsequent evaluated 2D BSHs were similar in every 5% or 12.5% estimation at coronal 25% 2D BSH levels. However, the amount of 3D BA-RSAs at four subsequent evaluated 25% 2D BSHs levels demonstrated a significant pattern of decrease for both maxillary and mandibular premolars (F test < 0.05, Table 1).

It is difficult to measure the exact amount of 3D BA-RSA clinically and radiographically. One goal of this study was to elucidate the proportion of 2D BSHs according to the amount of evaluated 3D BA-RSAs *in vitro* (Table 2, Fig. 2). From 3D BA-RSAs perspective, the proportion of 2D BSH can be accurately evaluated according to the coronal 15% 3D BA-RSAs levels for the maxillary and 10% 3D BA-RSAs levels for the mandibular premolars theoretically (Fig. 2, Table 2, H0). Moreover, after relating the data of root length, BSH % and BA-RSA%, it was 1.66 mm apical to the CEJ for the maxillary and 1.2 mm for the mandibular premolars that could be used to synchronize the BSH and BA-RSA levels. Different from the 2D’s viewpoint, the proportions of two subsequent 2D BSHs can be differentiated significantly judging by two subsequent 3D BA-RSAs analyses in all examined 5%, 12.5% and 25% levels (F test < 0.05, Table 2). These findings suggested that the amount of bone loss can be more accurately appraised by using 3D calculation than by which of 2D’s.

Maxillary premolar roots have a complex anatomy and retain a substantially proximal indentation below the CEJ^10^; and mandibular premolars show regular and less tapered roots, which might partially explain the some differences that were standardized according to the BSH or BA-RSA. Nevertheless, maxillary and mandibular premolars demonstrated a non-significant percentage difference at most evaluated BSH and BA-RSA levels (Tables 1 and 2).

The BA-RSA % at subsequent levels differed significantly for all four 25% BSHs, and higher BA-RSA % were observed on the coronal portion (Table 1). The alveolar bone loss appraised according to the 2D BSH or 3D BA-RSA measurement was thus inconsistent. When 25% coronal alveolar bone lost (at the 75% BSH level), the maxillary and mandibular premolars retained 68.27% and 67.33% BA-RSA, respectively. This implies that, when the premolar roots lost their coronal 25% bone attachment, as observed on a 2D periapical radiograph, more than 31% BA-RSA support was lost. Consequently, if premolar roots lost 50% 2D BSH, the tooth roots could lose approximately 60% BA-RSA support. Similar data were presented in a previous study that examined 9 mandibular premolars and reported that 61.5% RSA loss was associated with 50% attachment remaining^5^. By contrast, the present study reviewed both the maxillary and mandibular premolars and offered additional evidence for other surveyed levels. Sample size, participant ethnicity, methodology, and equipment contributed to some variation between this study and that of Levy^5^, such as mandibular premolar root length (14.5 ± 2.4 mm vs. 13.45 ± 1.47 mm) and RSA (258.7 ± 76.2 mm^2^ vs. 199.51 ± 26.33 mm^2^). Finally, 16.58% BA-RSA of maxillary premolar and 14.61% BA-RSA of mandibular premolar retained at 25% 2D BSH levels (Fig. 1). Previous studies have reported various total RSAs for premolars^6^,^11^,^12^,^13^,^14^,^15^,^16^. A wide range of tooth size, root anatomy, and diverse methods and applications may elucidate this dissimilarity. Thus, the ratio of BA-RSA to BSH for individual teeth were first measured and calibrated before statistical analysis was performed in this study. From 3D aspect, coronal 25% BA-RSA corresponded to the maxillary premolars 78.80% 2D BSH level and the mandibular premolars 79.67% BSH level. Similar to Nicholls^17^, our study showed that 59.3–59.8% 2D BSH represented 50% BA-RSA loss. Moreover, approximately 33% apical root length (35.33–36.43% BSH) implied 25% apical BA-RSA preservation (Fig. 2).

In a previous study, a coordinate measurement machine with a 0.4-mm diameter probe was used to accurately identify RSA in 8 premolars from simulated X-ray projections. The RSA of a tooth with a single root was evaluated with clinical accuracy from the projection data^7^. However, another study concluded that a reliable appraisal of the RSA and BSH ratio could not be determined from 2D linear or area data^8^. The inconsistent outcomes may be due to different sample sizes, appliances, methodologies, or statistical analyses^7^,^8^.

Inconsistent amount of alveolar bone loss appraised by 2D BSH or 3D BA-RSA measurement could be crucial for clinicians who place great emphasis on bone support or the crown–root ratio in determining a prognosis of an abutment tooth. However, the characteristics of the epithelium and gingival fibers play a critical role in periodontal health, and their impact on periodontal prognoses and tooth support require further attention. The status of periodontitis has been extensively evaluated using the concept of CAL. Approximately 1.0 mm of connective tissue attachment should be considered before offering a periodontal prognosis for the evaluated teeth^9^,^18^. When the current BA-RSA data are analyzed to elucidate the CAL theory, at least 1.0 mm of connective tissue attachment must be considered (epithelium attachment was excluded). Therefore, by interpreting the BA-RSA findings with the CAL concept, the authors assert that: When the periodontal probing CAL exceeds 3 mm (12.6–9.93 = 2.67 mm for maxillary premolars and 13.45–10.72 = 2.73 mm for mandibular premolars), the premolars present coronal 25% 3D RSA periodontal attachment and 4 mm 2D BSH detached. Correspondingly, it is about 30% coronal BSH loss could be shown on 2D periapical film (2.67 + 1 = 3.67 mm and 3.67mm/12.6 mm = 29% for the maxillary; 2.73 + 1 = 3.73 mm and 3.73mm/13.45 mm = 27% for the mandibular). A fair prognosis referring to the McGuire classification^3^,^4^ occurred. The occurrence of a periodontal loss of 5.13–5.38 mm probing CAL (12.60–7.47 = 5.13 mm for maxillary premolars, and 13.45–8.07 = 5.38 mm for mandibular premolars) may have indicated that a 50% 3D RSA of the teeth had lost its periodontal support and that the prognosis was poor. Comparatively, about 50% BSH loss (5.13 + 1 = 6.13 mm and 6.13mm/12.6 mm = 48.6% for the maxillary premolars; 5.38 + 1 = 6.38 mm and 6.38 mm/13.45 mm = 47.4% for the mandibular premolars) was shown on the periapical film. This BSH amount of approximately 50% could be correlated with the 40% apical BA-RSA preserved (41.24% for the maxillary premolars, and 39.15% for the mandibular premolars; Fig. 1). A McGuire’s questionable prognosis can be then correlated. Finally, only 25% apical RSA retained their periodontal attachment when the periodontal probing CAL exceeded 8 mm (12.6–4.47 = 8.13 mm for maxillary premolars, and 13.45–4.91 = 8.54 mm for mandibular premolars). In addition, more than 70% 2D BSH loss (8.13 + 1 = 9.13 mm and 9.13 mm/12.6 mm = 72% for the maxillary premolars; 8.54 + 1 = 9.54 mm and 9.54 mm/13.45 mm = 71% for the mandibular premolars) were shown on the periapical film. However, other factors, such as root concavities, root proximity, furcation involvement, tooth mobility, caries, abutment selection, tooth vitality, and root resorption, should be considered before assigning a premolar periodontal prognosis.

Vertical periodontal bone loss, including proximal intrabony defects, buccal/lingual dehiscence, and fenestration, may be mistaken for 3D bone-detachment with 2D bone support, causing some evaluated variation leading to inaccurate prognosis, thus limiting the referred effect of this study.

Although studies on integral teeth are uncommon, future studies on incisors, canines, and molars with a large sample size for statistical analysis are warranted.

## Conclusions

Under the limitation of this study, we concluded that approximately 1.2–1.7 mm 2D BSH and 10–15% 3D BA-RSA apical to CEJ for premolars may represent to each other. When premolars lost coronal 25%, 50%, 75% 2D BSH, approximately 70%, 40%, 15% 3D BA-RSA (68.3–67.3%, 41.2–39.2% and 16.6–14.6%) was preserved respectively. However, premolars lost 25%, 50%, 75% coronal 3D BA-RSA when approximately 79–80%, 59–60%, 35–36% 2D BSH remained respectively. The amount of 3D bone loss is therefore under assessed from 2D perspective for single-rooted premolars. The evaluated severity of periodontitis, assigned prognosis, and designed treatment plan for the teeth with moderate to severe periodontitis from 3D aspect are therefore dissimilar with which from 2D’s.

## Methods

### Samples and laser scanner

Thirty-six extracted and intact human maxillary and 35 mandibular single-rooted premolars were collected from patients with trauma, periodontitis, or from patients who had undergone orthodontic treatment in the Dental Department of Chang Gung Memorial Hospital (CGMH). This clinical study followed the Declaration of Helsinki and approved by the Medical Ethics Committee of Chang Gung Memorial Hospital. All methods were performed in accordance with the Taiwan Dental Association guidelines and regulations. All patients provided written informed consent.

The 3D spatial coordinates of the premolar morphology were obtained using a laser scanner (DentSCAN, Delcam, Birmingham, UK) with an accuracy of 0.02 mm. First, the apex of every premolar was fixed vertically on a fixture. A thin layer of white powder was sprayed uniformly onto the premolar surface for contrast during white-light scanning. The teeth were placed on a 360° rotating platform that was controlled using a computer. The teeth were irradiated using a light-emitting diode grating, and two charge-coupled device illumination lenses were used to capture images from different angles. A total of approximately 30,000–40,000 points with 3D point coordinates on the premolar surface were acquired over approximately 1 minute. Subsequently, the crown of each premolar was fixed on the fixture, and the scan was repeated to obtain the second point cloud.

According to the CEJ orientation, the two point clouds were aligned and merged into a single premolar model by using DentCAD software (Delcam, Birmingham, UK) to align the overlapping area of the point clouds. Finally, a stereo lithography format model was developed with approximately 15,000–20,000 fine triangle surface meshes, forming a 3D model of the premolars. The premolar root area was calculated as the sum of specific fine triangle areas by using Pro/ENGINEER software (PTC, Needham, MA, USA).

**Figure 3:**
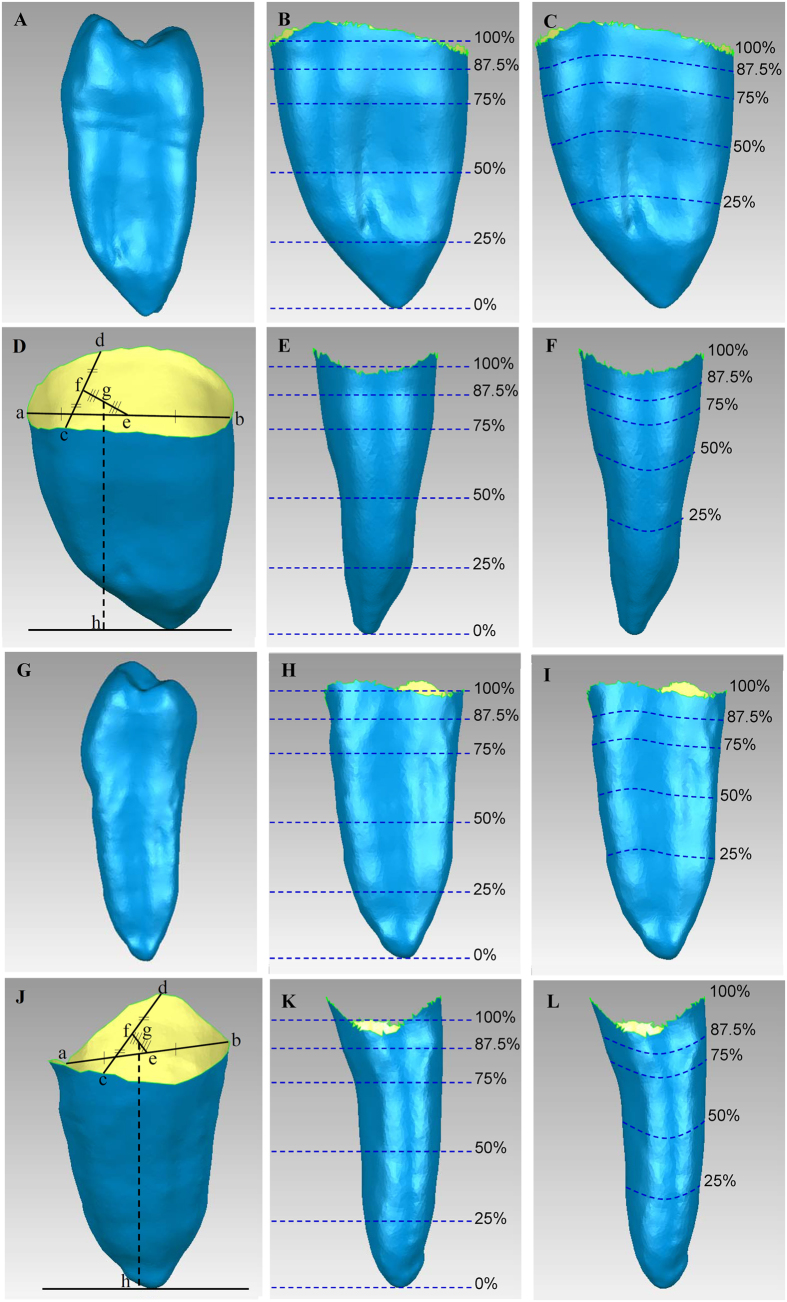
(**A–F**): Views and relative levels of BSH and BA-RSA of maxillary premolar. (**A**) View of the mesial surface of the laser-scanned whole premolar, (**B**) mesial view of the BSH levels, measured from the apex to the CEJ; (**C**) mesial view of the detected BA-RSA amounts, measured apicocoronally; (**D**) a,b: line connecting the buccal and lingual CEJ, c,d: line connecting the mesial and distal CEJ, e: midpoint of a,b, f: midpoint of c,d, g: midpoint of e,f, and g,h: root length; (**E**) buccal view of the appraised BSH levels, measured apicocoronally; (**F**) buccal view of the surveyed BA-RSA amounts, measured apicocoronally. (**G–L**): Views and relative levels of BSH and BA-RSA of mandibular premolar. (**G**) View of the mesial surface of the laser-scanned whole premolar (**H**) mesial view of the BSH levels, measured from the apex to the CEJ; (**I**) mesial view of the detected BA-RSA amounts, measured apicocoronally; (**J**) a,b: line connecting the buccal and lingual CEJ, c,d: line connecting the mesial and distal CEJ, e: midpoint of a,b, f: midpoint of c,d, g: midpoint of e,f and g,h: root length; (**K**) buccal view of the BSH levels, measured apicocoronally; (**L**) buccal view of the BA-RSA amounts, measured apicocoronally.

Root length is the distance from the apex to an average CEJ point (g,h in Fig. 3D and J), for which the CEJ point is defined as the midpoint of the two midpoints of the interproximal and buccolingual lines (f and e, respectively, in Fig. 3).

The explored 2D levels (95%, 90%, 87.5%, 85%, 80%, 75%, 50%, and 25% of the BSH) and 3D BA-RSA planes (95%, 90%, 87.5%, 85%, 80%, 75%, 50%, and 25% of the BA-RSA) were also calculated and analyzed using Pro/ENGINEER software (Fig. 3).

### Statistical Analysis

Maxillary and mandibular premolar groups’ statistics were calculated after each tooth calibration (comparing the proportion at evaluated BSH level to which amount of the BA-RSA, vice versa, for every individual tooth first before group’s statistical analysis) to avoid tooth size and root morphology bias. Significant differences between the samples were analyzed

A one-sample *t* test was used to compare the differences between the 3D BA-RSA and corresponding 2D BSH (*p* < 0.01):

H0, which is the variation between the BA-RSA and BSH, was |mean - x| ≤ 2% at the evaluated levels (where x = 95%, 90%, 87.5%, 85%, 80%, 75%, 50%, and 25%, respectively).

H1, which is the variation between the BA-RSA and BSH, was |mean - x| > 2% at the evaluated levels (where x = 95%, 90%, 87.5%, 85%, 80%, 75%, 50%, and 25%, respectively).

Paired *t* tests were used to explore the significant differences between 2 subsequently examined 3D BA-RSA (and 2D BSH) levels at 5% group (100–95%, 95–90%, 90–85%, 85–80% and, 80–75%), 12.5% group (100–87.5% and 87.5–75%), and 25% group (100–75%, 75–50%, 50–25%, and 25–0%), where *p* < 0.01.

Independent *t* tests were used to study the variations in 3D BA-RSA (and 2D BSH) between the maxillary and mandibular premolars (*p* < 0.05).

F tests were applied to examine the developing significance of BA-RSA or BSH in 5%, 12.5% and 25% groups (*p* < 0.05).

## Additional Information

**How to cite this article:** Hong, H.-H. *et al*. Decreased Amount of Supporting Alveolar Bone at Single-Rooted Premolars Is Under Estimated by 2D Examinations. *Sci. Rep.* **7**, 45774; doi: 10.1038/srep45774 (2017).

**Publisher's note:** Springer Nature remains neutral with regard to jurisdictional claims in published maps and institutional affiliations.

## Figures and Tables

**Table 1:** Comparison of the BA-RSA significance at the evaluated BSH levels.

BA-RSA % at various BSHs	Maxillary premolars (n = 36)			Mandibular premolars (n = 35)			Maxilla vs. Mandible
	Mean ± SE	*P* < 0.01	F test *p*	Mean ± SE	*p* < 0.01	F test *p*	*p* < 0.05
**5%**			0.084			0.077	
BA-RSA % at 100–95% BSH	6.33 ± 0.22%	=0.542		6.32 ± 0.25%	=0.118		0.986
vs. at 95–90% BSH	6.18 ± 0.13%			6.76 ± 0.13%			0.003**
BA-RSA % at 95–90% BSH	6.18 ± 0.13%	= 0.002**		6.76 ± 0.13%	=0.758		0.003**
vs. at 90–85% BSH	6.66 ± 0.12%			6.80 ± 0.09%			0.367
BA-RSA % at 90–85% BSH	6.66 ± 0.12%	=0.005**		6.80 ± 0.09%	=0.001**		0.367
vs. at 85–80% BSH	6.24 ± 0.10%			6.45 ± 0.10%			0.145
BA-RSA % at 85–80% BSH	6.24 ± 0.10%	=0.370		6.45 ± 0.10%	=0.268		0.145
vs. at 80–75% BSH	6.33 ± 0.09%			6.34 ± 0.07%			0.931
**12.5%**			0.785			0.342	
BA-RSA % at 100–87.5% BSH	15.90 ± 0.31%	=0.785		16.48 ± 0.29%	=0.342		0.163
vs. at 87.5–75% BSH	15.84 ± 0.19%			16.19 ± 0.19%			0.198
**25%**			<0.001			<0.001	
BA-RSA % at 100–75% BSH	31.73 ± 0.45%	<0.001***		32.67 ± 0.37%	<0.001***		0.118
vs. at 75–50% BSH	27.07 ± 0.40%			28.17 ± 0.39%			0.051
BA-RSA % at 75–50% BSH	27.07 ± 0.40%	<0.001***		28.17 ± 0.39%	<0.001***		0.051
vs. at 50–25% BSH	24.60 ± 0.31%			24.54 ± 0.24%			0.868
BA-RSA % at 50–25% BSH	24.60 ± 0.31%	<0.001***		24.54 ± 0.24%	<0.001***		0.868
vs. at 25–0% BSH	16.59 ± 0.47%			14.62 ± 0.39%			0.002**

Paired *t-*test for significant differences in BA-RSA at various BSH levels: *p < 0.05, **p < 0.01, ***p < 0.001.

Independent *t* test for maxilla vs. mandible: *p < 0.05, **p < 0.01, ***p < 0.001 F test for the significance of intra-group variances of 5%, 12.5% and 25% groups: *p < 0.05, **p < 0.01, ***p < 0.001.

**Table 2:** Comparison of the BSH significance at various BA-RSA levels.

BSH % at various BA-RSA amount	Maxillary premolars (n = 36)			Mandibular premolars (n = 35)			Maxilla vs. Mandible
	Mean ± SE	*p* < 0.01	F test *p*	Mean ± SE	*p* < 0.01	F test *p*	*p* < 0.05
**5%**			0.001			0.005	
BSH % at 100–95%BA-RSA	5.00 ± 0.29%	=0.065		4.88 ± 0.33%	=0.032		0.768
vs. at 95–90% BA-RSA	4.34 ± 0.13%			4.05 ± 0.12%			0.112
BSH % at 95–90%BA-RSA	4.34 ± 0.13%	=0.020		4.05 ± 0.12%	=0.279		0.112
vs. at 90–85% BA-RSA	3.86 ± 0.13%			3.81 ± 0.14%			0.814
BSH % at 90–85% BA-RSA	3.86 ± 0.13%	=0.859		3.81 ± 0.14%	=0.809		0.814
vs. at 85–80% BA-RSA	3.90 ± 0.12%			3.75 ± 0.15%			0.479
BSH % at 85–80% BA-RSA	3.90 ± 0.12%	=0.303		3.75 ± 0.15%	=0.751		0.479
vs. at 80–75% BA-RSA	4.11 ± 0.13%			3.83 ± 0.15%			0.181
**12.5%**			0.003			0.005	
BSH % at 100–87.5% BA-RSA	11.23 ± 0.32%	=0.003**		10.81 ± 0.36%	=0.005**		0.390
vs. at 87.5–75% BA-RSA	9.99 ± 0.14%			9.51 ± 0.16%			0.029*
**25%**			<0.001			<0.001	
BSH % at 100–75% BA-RSA	21.21 ± 0.29%	=0.001**		20.32 ± 0.36%	=0.255		
vs. at 75–50% BA-RSA	19.62 ± 0.29%			19.73 ± 0.27%			0.060
BSH % at 75–50% BA-RSA	19.62 ± 0.29%	<0.001***			<0.001***		0.761
vs. at 50–25% BA-RSA	23.84 ± 0.17%			19.73 ± 0.27%			0.761
				23.52 ± 0.17%			0.184
BSH % at 50–25% BA-RSA	23.84 ± 0.17%	<0.001***		23.52 ± 0.17%	<0.001***		0.184
vs. at 25–0% BA-RSA	35.33 ± 0.41%			36.43 ± 0.42%			0.064

Paired *t-*test for significant differences in BSH% at various BA-RSA levels: *p < 0.05, **p < 0.01, ***p < 0.001.

Independent *t* test for maxilla vs. mandible: *p < 0.05, **p < 0.01, ***p < 0.001 F test for the significance of intra-group variances of 5%, 12.5% and 25% groups: *p < 0.05, **p < 0.01, ***p < 0.001.
